# Questionnaire Review: Management Standards Indicator Tool

**DOI:** 10.1093/occmed/kqaf002

**Published:** 2025-07-14

**Authors:** Jonathan Houdmont

## A brief history

The Management Standards Indicator Tool (MSIT) was developed by the Health and Safety Executive (HSE) —the workplace health and safety regulator in Britain—for workers to report on the quality of their psychosocial work environment. Launched as part of the HSE’s Management Standards approach to supporting organizations in tackling work-related stress [1], the MSIT reflects a set of aspirational Standards covering key areas of work design that if not properly managed may be associated with impaired health and organizational effectiveness. Encompassing ‘the primary sources of stress at work’ [2], the Management Standards and associated MSIT are centred on seven psychosocial work environment characteristics, namely: demands, control, managerial support, peer support, role, relationships and change. The MSIT helps organizations assess the extent to which the Management Standards are met and identify where work design modifications may be warranted to support their achievement.

## Items and scoring details

The MSIT contains 35 items that assess the quality of the psychosocial work environment over the preceding 6-month period across the Management Standard areas: demands (eight items), control (six items), managerial support (five items), peer support (four items), role (five items), relationships (four items) and change (three items). Items are presented in the form of statements. For the first 23 items, respondents indicate on a scale of never (1) to always (5) the frequency with which a state is experienced (e.g. ‘I am clear what is expected of me at work), with negatively framed items (e.g. ‘I have unachievable deadlines’) reverse scored so that high scores indicate good psychosocial work environment quality. The remaining items require respondents to indicate their degree of agreement with a statement on a scale of strongly disagree (1) to strongly agree (5) (e.g. ‘I have some say over the way I work’), with negatively framed items (e.g. ‘I am subject to bullying at work’) reverse scored.

A mean score is generated for each standard with a score of 1 indicating poor psychosocial work environment quality while 5 indicates that the standard is being fully met. These calculations may be performed using an Excel-based Analysis Tool, though the performance of more advanced analysis involving, for instance, examination of linkages between psychosocial work environment quality and indices of health, requires statistical software.

A 25-item version of the MSIT was subsequently developed through the deletion of 10 items with a low factor loading [3]. The short MSIT has proven popular amongst researchers [4,5] owing to the reduction in time required for survey completion. However, it remains less familiar to practitioners because of its unavailability on the HSE website.

## Validity

The inter-item reliability (Cronbach’s alpha coefficient) for each area is consistently acceptable (>0.70) across studies. The short and full versions are widely used and offer similar concurrent validity with respect to emotional exhaustion and psychological distress [6].

## Key research

The MSIT was conceived to support organizations in the assessment of psychosocial work environment quality as part of the Management Standards approach. The extent to which it is utilized for this purpose is difficult to gauge, though some indication can be gleaned from the Chartered Institute of Personnel and Development’s 2023 Health and Wellbeing at Work survey which found that 20% of 438 surveyed organizations in the UK used the Management Standards [7].

Spearheaded by Kinman and colleagues [8,9], a key strand of research has centred on benchmarking psychosocial work environment quality against national general workforce norms. The gradual emergence of sector-specific reference data has allowed for the expansion of this approach to include comparisons against sector-specific normative values alongside those pertaining to the general national workforce. For instance, Smits and colleagues [10] compared the MSIT scores of autistic veterinary surgeons to those generated by a large-scale sector-wide survey of the veterinary profession [11] alongside UK workforce norms published for this purpose by Edwards and Webster [3] pertaining to public (*N* = 59 636) and (*N* = 7589) private sector workers. Edwards and Webster’s dataset represents a valuable benchmarking resource. Nevertheless, much has changed in the world of work since its publication in 2012 and the possibility exists that these data may fail to offer an accurate reflection of contemporary psychosocial work environment quality. Care should be applied in drawing comparisons with these reference values. In the absence of an updated normative dataset, organizations may prefer to compare their data to that drawn from contemporary sector-representative surveys (where available) or focus instead on gauging internal improvement based on repeat administration of the MSIT over time.

Most MSIT research has sought to establish the strength and direction of cross-sectional linkages between each standard and indices of mental health, with a particular focus on burnout, mental well-being and psychological distress [8,11,12]. These studies have consistently demonstrated the relevance of the psychosocial work environment dimensions encapsulated in the Management Standards to mental health. A smaller body of research has applied the same approach with indices of organizational effectiveness such as job performance [13], attendance behaviours [14] and turnover intentions [5,14]. This latter group of studies demonstrates that the relevance of a high-quality psychosocial work environment extends beyond workers’ health to that of the organization, and in doing so has contributed to the business case for the deployment of the Management Standards approach.

The MSIT’s restricted focus on a limited set of generic aspects of the psychosocial work environment renders it insensitive to context-specific work characteristics that may meaningfully contribute to the determination of workers’ health and wellbeing. For instance, the application of the MSIT in Bartram and colleagues [11] study of veterinary surgeons allowed for the quantification of psychosocial factors that apply to workers across roles, organizations and sectors, yet important psychosocial factors embedded in the specific job role—such as euthanasia—remained unexplored. As such, it is not uncommon to administer a set of role- or sector-specific survey items alongside the MSIT to obtain a rich understanding of psychosocial work quality sufficient to inform interventions that address local needs. Two decades since its publication, the MSIT arguably requires a refresh to reflect changes in ways of working brought about by technological developments, the coronavirus disease-2019 pandemic and evolving cultural norms. Work is underway in this regard; for instance, the Home and Hybrid Working Stress Indicator Tool developed by researchers at the University of Hull supplements the MSIT with items exploring psychosocial aspects of remote working [15].

## Data Availability

The MSIT (in 19 languages), user manual and analysis tool are available free of charge on the HSE website at https://www.hse.gov.uk/stress/standards/downloads.htm.
